# Covid-19 and Confinement: Effect on Weight Load, Physical Activity and Eating Behavior of Higher Education Students in Southern Morocco

**DOI:** 10.5334/aogh.3144

**Published:** 2021-01-06

**Authors:** Mohamed Boukrim, Majdouline Obtel, Jalal Kasouati, Abderrahmane Achbani, Rachid Razine

**Affiliations:** 1 Laboratory of Biostatistics, Clinical Research and Epidemiology, Faculty of Medicine and Pharmacy, Mohammed V University, Rabat, Morocco; 2 Higher Institute of Nursing and Health Techniques, Laayoune, Morocco; 3 Social Medicine Laboratory (Public Health, Hygiene and Preventive Medicine), Faculty of Medicine and Pharmacy, Mohammed V University, Rabat, Morocco; 4 Cell Biology and Molecular Genetics Laboratory, Faculty of Science, Ibn Zohr University, Agadir, Morocco

## Abstract

**Background:**

Pandemic confinement of COVID-19 may influence dietary behaviors and physical activity, and increases the risk of stress, especially among adolescents. This increases the subsequent risk of degenerative diseases such as obesity, diabetes, cardiovascular disease, etc., which can lead to a higher risk of death. This study aims to evaluate the effect of confinement on the weight load, physical activity and dietary behavior of higher education students during the period of confinement.

**Methods:**

Data was collected by an anonymous online questionnaire with 406 students. Physical activity was evaluated with the International Physical Activity Questionnaire. A reference score of the National Nutrition and Health Program (PNNS-GS) was used to determine the dietary habits. Stress appreciation was assessed by using a psychological instrument known as the “Perceived Stress Scale” provided by Mind Garden.

**Results:**

More than a quarter of the students were overweight or obese. During the confinement of COVID-19, most of the students suffered from nutritional disorders, only one-third were moderately physically active, and the majority of students were at risk of stress. Multivariate analysis showed that the concept of threat of Stress increases the risk of weight gain at a risk level of 2.4 [95% CI 1.09–5.43], low physical activity increases the risk level to 1.9 [95% CI 1.18–3.04]. However, a balanced diet is protective against the occurrence of weight gain (ORa = 0.30, [95% CI 0.15–0.61]).

**Conclusion:**

The study showed that confinement appeared to contribute to weight gain and those students were more sedentary than active with unhealthy eating behaviors. Understanding these behaviors during COVID-19 confinement will help public health authorities implement future policies on recommendations when new pandemics arrive and confinement policies are implemented.

## INTRODUCTION

University students, who are young adults, are going through a transition period during which it is essential to acquire good eating behaviors that can simultaneously influence current health status and adult predisposition to diseases such as obesity, diabetes, cardiovascular pathologies, etc. Indeed, many studies have described a change in students’ eating habits, with a decrease in physical activity levels, insufficient and poor quality sleep and high stress levels. These changes may be associated with weight gain [1]. In Canada, one study showed that 18.7% of students in a Quebec university were overweight, 57% of students were considered inactive and most suffered from eating disorders [2].

In addition, the world is currently experiencing a pandemic related to the spread of the SARS-CoV-19 virus, which is a large family of viruses that cause illnesses ranging from the common cold to more serious affections. A new strain of coronavirus that had not yet been detected in humans has been named “COVID-19 virus [3].”

This infection remains benign or even asymptomatic in 80% of cases. In 15% of cases, hospitalization with oxygen therapy is necessary and 5% require intensive care management with respiratory assistance and hemodynamic support [4]. The related disease was declared a pandemic by the World Health Organization (WHO) in March 2020 [5]. This constituted a novel situation in all aspects (health, economic and social). In the absence of effective vaccine and treatment, and in response to the pandemic, public health recommendations and government measures have imposed closures and restrictions. While these restrictions help reduce the rate of infection, they have negative effects by limiting participation in normal daily activities, physical activity, travel and access to many forms of exercise (e.g., closed gyms, no group gatherings, social/physical distance). This can promote the appearance of sedentary behaviors and eating disorders that could have a negative impact on health status, including an increased risk of chronic diseases such as hypertension, diabetes, obesity and overweight….

In the absence of physical activity, the musculoskeletal system is the first to be affected due to the loss of 12% of muscle mass and the decrease in bone density is more insidious and bone loss is estimated to be around 1% after one month of inactivity mainly in the load-bearing bones, lumbar spine and especially the femoral head region [6,7].

On the mental level, the current situation of confinement is a source of stress which can lead to neurodegenerative diseases with disorders of memory and spatial orientation that could be delayed or prevented by physical activity [8,9].

Also, the confinement due to the COVID-19 pandemic may influence dietary profiles, especially those of adolescents and young adults, who are very likely to develop poor eating habits and consequently the subsequent risk of degenerative diseases such as obesity, diabetes, cardiovascular pathologies, and so on.

Furthermore, the World Health Organization and the Spanish Academy of Nutrition and Dietetics indicate that a healthy diet can contribute to the prevention and treatment of disease since good nutrition plays a central role in the development and maintenance of the immune system [10].

Morocco, like other countries, decreed on 24/03/2020 a state of public health emergency throughout the national territory in order to cope with the spread of the corona virus covid-19 [11]. The objective of this study is to estimate the prevalence of weight load and to evaluate the effect of confinement on the physical activity and dietary behavior of higher education students during the period of confinement.

## MATERIALS AND METHODS

This is a cross-sectional observational study of 406 students at five public institutions of higher education with students from the Souss region and the three southern regions. These institutions are located in the capital of the provinces of Agadir Ida-outanane and Laayoune.

Data were collected from April 01 to June 10, 2020 (end of the confinement).

Collection was done through an online questionnaire covering sociodemographic and student data, physical activity, diet and measurement of perceived stress levels.

To assess dietary habits, a suitability score derived from the Guideline Score of the French National Nutrition and Health Program (PNNS-GS) was used. It is composed of simple questions on the frequency of consumption of major food groups (fruits and vegetables, starchy foods, meat and poultry, dairy products…). The adherence of adults to the PNNS recommendations was measured by the PNNS-Guideline reduced score (PNNS-GSR).

The general principle is to award one point when the recommendation is reached, intermediate points are awarded when the recommendation was only partially reached (0.5). However, points were subtracted (-1) when exceeding the recommended intakes is considered to be detrimental to health [12].

For data analysis, the scores were divided into three slices [13]:

**Table:** 

Food slices	Score PNNS-GS
T1	Score < 2.5
T2	2.5 ≤ score < 5
T3	Score ≥ 5

Physical activity was assessed using the International Physical Activity Questionnaire (IPAQ), Short Version [14]. The short version includes seven physical activity items providing information on time spent walking, vigorous and moderate intensity activity and sedentary activity [15]. The choice of this questionnaire is to have a common instrument that can be used to obtain internationally comparable data on health-related physical activity.

The body mass index (BMI) was calculated by the following formula: BMI (kg/m^2^) = weight (in kg)/height^2^ (in m^2^). The BMI is recognized as an international criterion for assessing weight load. According to the WHO thresholds, overweight is defined as a BMI equal to or greater than 25 kg/m^2^ and obesity as a BMI equal to or greater than 30 kg/m^2^ [16]. Due to the confinement period and the cessation of face-to-face classes, information on anthropometric data (height and weight) was reported by the students. The degree of appreciation of stressful situations was measured by the psychological instrument “Perceived Stress Scale” hosted by Mind Garden [17].

The data was encoded and analyzed using SPSS version 13. Qualitative variables were described in terms of headcount and percentage and then compared using chi-square and exact Fisher tests according to the conditions of application of each. Quantitative variables were expressed as means and standard deviation (SD).

To examine the relationship between several variables simultaneously and to eliminate confounding factors, we performed a multivariate analysis by conditional logistic regression, which allows us to estimate the different Odds-ratios adjusted with their confidence intervals. The variables inserted in the model to compose the predictor were chosen according to their clinical relevance, their statistical link to the dependent variable, at the 20% threshold, in the univariate analysis (p-value of the Wald test).

## CONFIDENTIALITY OF DATA AND CONSENT TO PARTICIPATE

The study was conducted with the free and informed consent of participants. Survey participants were assured that all data would be used only for research purposes. Participant responses are anonymous and confidential. In addition, participants were given the opportunity to end their participation in the study and leave the questionnaire at any time prior to submission. Responses were saved only by clicking on the “send” button provided for this purpose. By completing the questionnaire, participants acknowledged that they had consented to voluntarily participate in the study.

## RESULTS

More than a quarter of the students (26.4%) were overweight, while almost two-thirds were of normal body weight. The study revealed that the mean BMI was 24.26 Kg/m^2^ ± 3.78 in a population with an average age of 20.10 years ± 1.36 with an average height and weight of 1.65 m (±0.09) and 65.57 Kg (±10.59) respectively. The study revealed that the mean BMI was 24.26 Kg/m^2^ ± 3.78 in a population with an average age of 20.10 years ± 1.36 with an average height and weight of 1.65 m (±0.09) and 65.57 Kg (±10.59) respectively. Three-fourths of the students were female, one-third were of Amazigh ethnicity, and most lived in urban areas (***Table 1***).

**Table 1:** Distribution of students according to socio-demographic and student characteristics.


VARIABLES	MEAN ± SD**	n	%

**Age**	20.10 years ± 1.36		

**Level of formation**			

1st year		138	34.00

2nd year		128	31.50

3rd year		140	34.50

**Gender**			

Female		302	74.40

Male		104	25.60

**Ethnicity**			

Arabic		189	46.60

Amazigh		133	32.80

Sahrawi		84	20.70

**Residence**			

Urban		358	88.20

Rural		27	6.70

Suburban		21	5.20

**Parental Education Level**			

Illiterate		110	27.10

Primary		69	17.00

Secondary		108	26.60

Superior		119	29.30

**Parental Income**			

Less than 273.54 USD*		86	21.20

[273.54–547.07]		133	32.80

[547.07–820.61]		68	16.70

[820.61–1094.14]		67	16.50

More than 1094.14		52	12.80

**Marital Status**			

Single		394	97.00

Married		9	2.20

Divorced		2	0.50

Widower		1	0.20

**BMI in Kg/m^2^**	24.26 ± 3.78		

Meager		33	8.10

Normal		265	65.30

Overweight		88	21.70

Obesity		19	4.70


* USD: United States Dollar. ** SD: Standard Deviation.

***Table 2*** presents the consumption frequencies of the different food groups that are the subject of the numerical benchmarks in the PNNS. Overall, 48.28% (including 53% female) of the participants could be considered small consumers of fruits and vegetables (less than 3.5 portions/day). In contrast, only 43% of participants ate at least five fruits and vegetables daily. A total of 86.45% (3/4 of whom were female) of the respondents consumed starchy foods at least three times a day. Only 18.47% of participants met the PNNS benchmark for dairy products, while the majority (76.11%, 3/4 of whom were female) ate dairy products less than 2.5 times a day. Nearly half of the participants (45.57%, 3/4 of whom were female) consumed food from the “meat, poultry, eggs” group once or twice a day and 44.58% exceeded this criterion. In addition, more than half of the participants (52% of whom three quarters were female) ate less than twice a week fishery products. As for the consumption of sweet products, more than half (52%, of which three quarters were female) consumed them once or twice a week. Overall, more than two thirds of the students had an average nutritional score (T2), of which more than 60% had a weight load. Moreover, among the students who were overweight (BMI ≥ 25 Kg/m^2^), more than 93% had an unbalanced diet (***Table 3***).

**Table 2:** Frequency of consumption of food groups by gender.


VARIABLES	FEMALE N = 302		MALE N = 104		TOTAL N = 406	
		
	n	%	n	%	N	%

**Consumption of Fruits and Vegetables**						

[0–3.5] times per day	150	49.67	46	44.23	196	48.28

[3.5–5] times per day	19	6.29	17	16.35	36	8.87

[5–7.5] times per day	131	43.38	41	39.42	172	42.36

≥7.5 times per day	1	0.33	0	0	1	0.25

**Starch Consumption**						

[0–1] times per day	6	1.99	0	0	6	1.48

[1–3] times per day	35	11.59	14	13.46	49	12.07

[3–6] times per day	144	47.68	48	46.15	192	47.29

≥6 times per day	117	38.74	42	40.38	159	39.16

**Consumption of Milk and Dairy Products**						

[0–1] times per day	41	13.58	17	16.35	58	14.29

[1–2.5] times per day	195	64.57	56	53.85	251	61.82

[2.5–3.5] times per day	50	16.56	25	24.04	75	18.47

≥3.5 times per day	16	5.3	6	5.77	22	5.42

**Consumption of Meat, Poultry and Eggs**						

0 times per day	33	10.93	7	6.73	40	9.85

[0–1] times per day	0	0	0	0	0	0

[1–2] times per day	141	46.69	44	42.31	185	45.57

≥2 times per day	128	42.38	53	50.96	181	44.58

**Consumption of Fish and Seafood Products**						

<2 times/week	161	53.31	49	47.12	210	51.72

≥2 times/week	141	46.69	55	52.88	196	48.28

**Consumption of sweet products**						

<1 times per day	127	42.05	47	46.15	174	43.1

[1–2] times per day	163	53.97	50	48.08	213	52.46

≥2 times per day	12	3.97	7	6.73	19	4.68


**Table 3:** Distribution of participants by nutritional score and weight status.


NUTRITIONAL RINGS	BMI ≥ 25 KG/M^2^	BMI < 25 KG/M^2^	P-VALUE
	N (%)	N (%)	

T1	35 (16.20%)	46 (24.20%)	**0.0001**

T2	167 (77.30%)	109 (57.40%)	

T3	14 (6.50%)	35 (18.40%)	


***Table 3*** illustrated that 77% of the participants with a weight load had an average diet close to the PNNS norms and that the weight load is related to the nutritional status of the students (p < 0.0001).

***Table 4*** revealed that weight load is related to the intensity of physical activity and the threat of stress (p < 0.006 and p < 0.008). Only 36% of the participants with a weight load carried out moderate intensity physical activity while two thirds carried out low intensity physical activity. The majority of students at risk of stress were overweight.

**Table 4:** Distribution of Participants by Intensity of Physical Activity and Threat of Stress.


VARIABLES	WEIGHT LOAD (BMI ≥ 25 KG/M^2^)				P-VALUE

INTENSITY OF PHYSICAL ACTIVITY	YES		NO		
	
	n	%	n	%	

Low	139	64	145	76	<0.006

Moderate	77	36	45	24	

Intense	0	0	0	0	

**Threat of stress**					

Yes	189	87.50	180	90.90	

No	27	12.50	10	9.10	0.008


To highlight the factors associated with weight load, the BMI values were split into two dichotomous variables (weight load if BMI ≥ 25 Kg/m^2^ and no weight load if BMI < 25 Kg/m^2^). The results of the simple binary logistic regression analysis showed that the weight load is related (p < 0.05) to gender, personal expenses below 273.54 USD per month, threat of stress, low physical activity and that a balanced diet is a protective factor (p < 0.05). On the other hand, it is not associated with the consumption of meat, seafood, starchy and sweet products (***Table 5***).

**Table 5:** Simple binary logistic regression analysis between weight load and participant characteristics.


VARIABLES	n (%)	OR	IC 95%	P

**Sex**				

Female	302 (74.38%)	4.20	[2.58–6.85]	0.0001

Male	104 (2562%)	1		

**Expenses less than 273,54 USD**				

Yes	296 (72.91%)	1.53	[0.99–2.35]	0.057

No	110 (27.09%)	1		

**Menace of stress**				

Yes	369 (90.87%)	2.571	[1.21–5.46]	0.014

No	37 (10.11%)	1		

**Low Physical Activity**				

Yes	284 (69.95%)	1.785	[1.15–2.76]	0.009

No	122 (30.05%	1		

**Balanced diet**				

Yes	49 (12.07%)	1		0.0001

No	357 (87.93%)	0.31	[0.16–0.59]	

**Sleep duration (7h–8h)**				

Yes	230 (56.65%)	0.936	[0.63–1.39]	0.743

No	176 (43.35%)	1		


In the simple binary logistic, the main variables that were significantly associated with the occurrence of weight gain at the 0.2 threshold were: gender, personal expenditure less than 273.54 USD, stress threat, low physical activity and balanced diet. In multiple binary logistic regression analysis, the top-down introduction of the independent variables revealed significant associations between several predictive factors and the weight load in the study population during the confinement period. The concept of threat of stress increases the risk of weight load at a risk level of 2.4 [95% CI 1.09–5.43]. Low physical activity is a higher risk factor for the occurrence of weight gain, increasing the risk level to 1.9 [95% CI 1.18–3.04]. On the other hand, a balanced diet ORa = 0.30, [95% CI 0.15–0.61] seems to play a protective role against the occurrence of weight gain. However, it is not associated with personal expenditure of less than 273.54 USD per month (***Table 6***).

**Table 6:** Results of the multiple binary logistic regression analysis.


Variables	n	(%)	OR ADJUSTED	IC À 95%	P-VALUE ADJUSTED

**Sex**					

Female	302	(74.38%)	1		0.0001

Male	104	(2562%)	0.243	[0.146–0.40]	

**Threat of stress**					

Yes	369	(90.87%)	2.43	[1.09–5.43]	0.031

N**o**	37	(10.11%)	1		

**Low Physical Activity**					

Yes	284	69.95%	1.90	[1.18–3.04]	0.008

No	122	30.05%	1		

**Balanced diet**					

Yes	49	12.07%	0.30	[0.15–0.61]	0.001

No	357	87.93%	1		

**Expenses less than** 273,54 USD					

Yes	296	72.91%	0.82	[0.50–1.34]	0.42

No	110	27.09%	1		


## DISCUSSION

A total of 406 students were involved in the study, divided into two groups: females (n = 302) and males (n = 104), with a sex ratio of 2.9 in favor of the female population. The study revealed that the average BMI was 24.26 Kg/m^2^.

The study found that more than a quarter of the students were overweight (21.7%) or obese (4.7%) and two thirds were of normal weight, which was consistent with the results of other studies [18]. We suppose that confinement of the students for three months appears to be the cause of the weight gain since the students were deprived of their normal activities and physical activity in gyms or outside.

More than three-quarters of the overweight participants scored close to the PNNS standards. Only one-third of the weight-bearing participants were moderately active, while two-thirds were moderately active. The majority of students at risk of stress were loaded.

Several factors could explain this weight gain. Studies have shown that women are more likely to be overweight or obese [19]. Our results showed that 79% of female students were overweight.

In addition, confinement, limitation of normal activities and reduced physical activity due to confinement appear to contribute to weight gain. Indeed, a study of young people in prison showed that in three months of follow-up, 66% developed overweight or obesity [20]. In addition, during confinement the population was deprived of participation in normal daily activities, physical activity and gatherings (social/physical distancing). These facts could contribute to the risk of increased weight gain. To confirm this finding, it is relevant to evaluate the same variables in the same population after confinement.

Our study found that the majority of students were at risk of stress according to the Perceived Stress Scale, and more than half of them had a weight load (p < 0.05). Students were subject to stress caused, on the one hand, by difficulties in adapting to university and the new responsibilities of young adults and [21], on the other hand, by confinement, since students were more worried and anxious [22]. Pedagogical factors (possibility and modalities of exams, internships, success and value of diplomas), economic factors (job loss) and health factors (nature of the disease and its evolution) [23]. In addition, increased stress, anxiety and boredom on a daily basis during the pandemic and during containment would have contributed to higher energy intake, sleep disturbances and less exercise [24].

As for sleep disturbance, 38.2% of the respondents suffered from it and more than half of them were overweight. This disorder affected more girls than boys and this result corroborated that of Mestaghanmi in Casablanca in 2019 [19]. In addition, sleep disorders in students can have both physical and psychological effects; sleep deprivation affects cognitive performance throughout the week. Furthermore, it has been shown that people who are obese or overweight sleep an average of 16 minutes less per day than those of normal weight [25,26].

Sport contributes to the well-being of individuals and physical and mental health are guaranteed for athletes. Athletes also perceive that their health is excellent. Indeed, regular physical activity contributes to subjective well-being and overall quality of life, by acting on the factors that intervene in different dimensions (lower stress levels, satisfaction with the body, satisfaction through active participation in social life) [27]. In this study, almost 70% of the students were engaged in low-intensity physical activity, of which almost two-thirds were weight-bearing (p = 0.006) and 30% were engaged in moderate-intensity physical activity. Indeed, a study in France on the sports habits during confinement showed that 48.4% did less during this period and especially outside their home [28,29]. Similarly, a survey conducted by the French Federation of Physical Education and Voluntary Gymnastics showed that 59% of people practiced less physical activity than before confinement [28]. In addition, the Canadian study showed that 34% of adults felt that they were less physically active during confinement, with the decrease being greater among those who did not meet the WHO minimum physical activity recommendations [30]. Similarly, a study conducted to evaluate the effect of home confinement due to COVID-19 on dietary behavior and physical activity showed a decrease in physical activity during this period and that one third of the students were not physically active [22,31].

Adequate nutrition is considered a potential health factor in the early stages of life and adolescence [32]. Furthermore, confinement influences lifestyle, especially diet and physical activity [33]. In this study, young adults suffered from eating disorders. Indeed, the study showed that the majority of the participants had an unbalanced diet, almost all of whom had a weight load (p < 0.0001) which could be explained by a low consumption of fruit and vegetables, dairy and seafood products and a high consumption of meat and sweet products [13]. These results showed that confinement could lead to unhealthy food consumption patterns [31]. In addition, women were found to consume these foods more than men. This is confirmed by observational studies on nutritional behaviors and gender differences [34].

## CONCLUSION

Our results provided a first description of the weight load and dietary trends of adolescents in southern Morocco during the COVID-19 pandemic that could have a subsequent impact on their health. Also, these students were engaged in low-intensity activity probably due to the boredom and stress produced by the COVID-19 confinement.

These results are particularly pertinent because the experiences of previous epidemics have shown that it is necessary to maintain optimal nutrition at the individual and collective levels in order to improve the physical and mental health of the population. In this sense, it is necessary to know the dietary habits of each country to encourage a healthy lifestyle after the confinement or development of future responses to similar pandemics.

In addition, the study showed the positive association between sex, diet, physical activity and stress. Therefore, it is important to conduct further large-scale studies that will analyze the psychological impact and lifestyle (diet and physical activity) in students in order to encourage them to adopt healthy behaviors, especially after this period of confinement. Understanding these behaviors during COVID-19 confinement will help public health authorities implement future policies on recommendations when new pandemics arrive and confinement policies are implemented.
